# Detection of tumor *ALK* status in neuroblastoma patients using peripheral blood

**DOI:** 10.1002/cam4.414

**Published:** 2015-02-04

**Authors:** Valérie Combaret, Isabelle Iacono, Angela Bellini, Stéphanie Bréjon, Virginie Bernard, Aurélien Marabelle, Carole Coze, Gaelle Pierron, Eve Lapouble, Gudrun Schleiermacher, Jean Yves Blay

**Affiliations:** 1 Centre Léon-Bérard, Laboratoire de Recherche Translationnelle28 rue Laennec, 69373, Lyon Cedex 08, France; 2 INSERM U830, Laboratoire de Génétique et Biologie des Cancers, Equipe Recherche Translationnelle en Oncologie Pédiatrique et Département d'Oncologie Pédiatrique, Institut Curie26 rue d'Ulm, 75248, Paris Cedex 05, France; 3 Plateforme de séquençage ICGEX, Institut Curie26 rue d'Ulm, 75248, Paris Cedex 05, France; 4 Centre Léon-Bérard, Institut d'Hématologie et d'Oncologie Pédiatrique28 rue Laennec, 69373, Lyon Cedex 08, France; 5 Aix-Marseille University et APHM, Hôpital d'Enfants de La Timone, Service d'Hématologie-Oncologie Pédiatrique13385, Marseille Cedex 05, France; 6 Institut Curie, Unité de Génétique Somatique26 rue d'Ulm, 75248, Paris Cedex 05, France

**Keywords:** *ALK* mutation, cell-free DNA, ddPCR, neuroblastoma

## Abstract

New protocols based on ALK-targeted therapy by crizotinib or other ALK-targeting molecules have opened for the treatment of patients with neuroblastoma (NB) if their tumors showed mutation and/or amplification of the *ALK* gene. However, tumor samples are not always available for analysis of *ALK* mutational status in particular at relapse. Here, we evaluated the *ALK* mutational status of NB samples by analysis of circulating DNA, using the droplet digital PCR (ddPCR) system. ddPCR assays was developed for the detection of *ALK* mutations at F1174 and R1275 hotspots found in NB tumors and was applied for the analysis of circulating DNA obtained from 200 *μ*L of serum or plasma samples collected from 114 patients with NB. The mutations F1174L (exon 23 position 3520, T>C and position 3522, C>A) and the mutation R1275Q (exon 25 position 3824, G>A) were detected in circulating DNA. The sensitivity of our test was 100%, 85%, and 92%, respectively, and the specificity was 100%, 91%, and 98%, respectively. In conclusion, the assay that we have developed offers a reliable, noninvasive blood test to assess *ALK* mutational status at F1174 and R1275 hotspots and should help clinicians to identify patients showing an *ALK* mutation in particular when no tumor tissue is available.

## Introduction

Neuroblastoma (NB), the most frequent extracranial solid tumor of childhood, is characterized by wide clinical variability, with possible cellular maturation or spontaneous tumor regression on the one hand, or aggressive clinical behavior with rapid progression despite intensive therapeutic approaches on the other hand. The International Neuroblastoma Risk Group (INRG) currently stratifies patients into very low, low, intermediate, or high-risk categories based upon well-defined prognostic factors 1. These include patient's age at diagnosis, disease stage, histopathological diagnosis, and MYCN oncogene status. Furthermore, the presence of several genetic copy number alterations observed recurrently in NB tumors (deletion of 1p, 3p, 4p, and 11q and/or gain 1q, 2p, and 17q) have been shown to be of prognostic impact 2. Despite the extensive description of copy number alterations in NB, few single gene alterations have been shown to be driver mutations in NB oncogenesis. Activating mutations of the ALK tyrosine kinase receptor have been shown to occur in ∽8–10% of cases at diagnosis. To date, over 50 different *ALK* mutations affecting 12 different AA residues have been described, with two major hotspots at position 1174 and 1275 3–7. These two hotspots are involved in 70% of *ALK* mutations 8. In vitro and in vivo NB models studies have indicated the potential usefulness of ALK inhibitors in the presence of an activating *ALK* mutation 9–11 and a phase 1/2 study of crizotinib, a dual ALK/MET inhibitor, supports further investigation of efficacy in the subset of NB harboring *ALK* mutations 12.

The ddPCR (droplet digital PCR) is a highly sensitive recently developed technology 13, enables the absolute quantitation of nucleic acids in a sample and allows the detection of rare mutant alleles and the identification of mutational status of a patient's cancer after assessment of circulating tumor DNA (ctDNA) in cell-free plasma 14.

Few years ago, we and others have demonstrated the presence of circulating MYCN sequences in the peripheral blood of patients with NB 15–19. Based on these observations and as mutations of the ALK tyrosine kinase domain constitute an important potential therapeutic target, a program to identify the presence of *ALK* mutation in the peripheral blood of NB patients showing a high risk of relapse 20 was initiated: the F1174 and R1275 hotspots of *ALK* gene were investigated using the ddPCR system.

## Material and Methods

### Patient population and samples

Patients diagnosed with NB were enrolled in the study after obtaining informed consent from their parents, according to national law. This study was authorized by the ethics committees “Comité de Protection des Personnes Sud-Est IV,” L07-95 and L12-171, as well as “Comité de Protection des Personnes de Paris Ile de France I” ref 08-11728. Serum or plasma from 114 patients with NB was assayed by ddPCR. For a majority of cases, the *ALK* status of the tumor was defined previously by Sanger sequencing (exons 23–25). Samples (serum, plasma, and tumors) were obtained from patients classified as stage 2 (*n* = 1, case with MYCN amplification), stage 3 (*n* = 16 whose six cases showed a MYCN amplification), and stage 4 (*n* = 97) according to the INSS classification 21. All of them were collected at diagnosis except for one obtained at relapse. Serum or plasma samples were prepared by centrifugation of blood at 700 g for 10 min followed by careful aliquoting and freezing at −80°C. The storage of samples was performed 1–24 h after the collection.

### DNA isolation

Tumor DNA and normal DNA were extracted from frozen NB and lymphocytes samples using phenol chloroform, and circulating DNA was extracted from 200 *μ*L of plasma or serum using the QIAmp DNA microkit (Qiagen, Courtaboeuf, France) according to the plasma protocol recommended by the manufacturer.

### Sequencing of tumor and normal DNA

Sanger sequencing was performed on 101 tumor DNA samples. All of them contained more than 50% of tumor cells as confirmed by pathological analysis. After amplification of *ALK* exons 23–25, PCR products were purified with Illustra Exostar (GE Healthcare Europe GmbH, Velizy-Villacoublay, France) for 15 min at 37°C and 15 min by 80°C. A sequencing reaction was set up with 1 *μ*l of purified PCR products and the BigDye® Terminator v1.1 Cycle Sequencing Kit (Life Technologies, Saint Aubin, France) following the manufacturer's instructions. The purification of the DNA sequencing reactions was performed by precipitation with ethanol and washing with 70% ethanol for removing nonincorporated BigDye® terminators and salts. DNA was dissolved in 30 *μ*L of ultrapure water and incubated for 10 min with agitation of 900 rpm. Sequencing analyses were carried out on the 48 capillary 3730 DNA Analyzer (Life Technologies). The primers used are described in Table1. To define if the mutation detected on tumor DNA samples was germline or somatic *ALK* mutations, the normal DNA samples obtained from lymphocytes of NB patients showing *ALK* mutations were studied.

**Table 1:** Primer sets and probes used for PCR in this study

Primers for Sanger sequencing		
ALK-23F	Primer	GCC-CAG-ACT-CAG-CTC-AGT-TA
ALK-23R	Primer	CAT-CCT-TGC-TCC-TGT-CCT-TG
ALK-ex25F	Primer	CCT-AGT-GAT-GGC-CGT-TGT-ACA-C
ALK-ex25R	Primer	GTA-CCA-GGA-GAT-GAT-GTA-AGG-GAC-AAG
Primers and probes for ddPCR		
ALK-1275-M	Probe	6-FAM-TTC-GGG-ATG-GCC-CAA-GAC-AT-MGB
ALK-1275-W	Probe	VIC-TTC-GGG-ATG-GCC-CGA-GAC-AT-MGB
ALK-F1174L3522-M	Probe	6FAM-TCT-CTG-CTC-TGC-AGC-AAA-TTA-AAC-C-MGB
ALK-F1174L3522-WT	Probe	VIC-TCT-CTG-CTC-TGC-AGC-AAA-TTC-AAC-C-MGB
ALK-F1174L3522-F	Primer	GCC-CAG-ACT-CAG-CTC-AGT
ALK-F1174L3522-R	Primer	CCC-CAA-TGC-AGC-GAA-CAA-T
ALK-1275-F	Primer	GTC-CAG-GCC-CTG-GAA-GAG
ALK-1275-R	Primer	GGG-GTG-AGG-CAG-TCT-TTA-CTC

The ALK F1174L3520 probes and primers were purchased from Biorad and the sequences were not known.

### ddPCR

**Figure 1:**
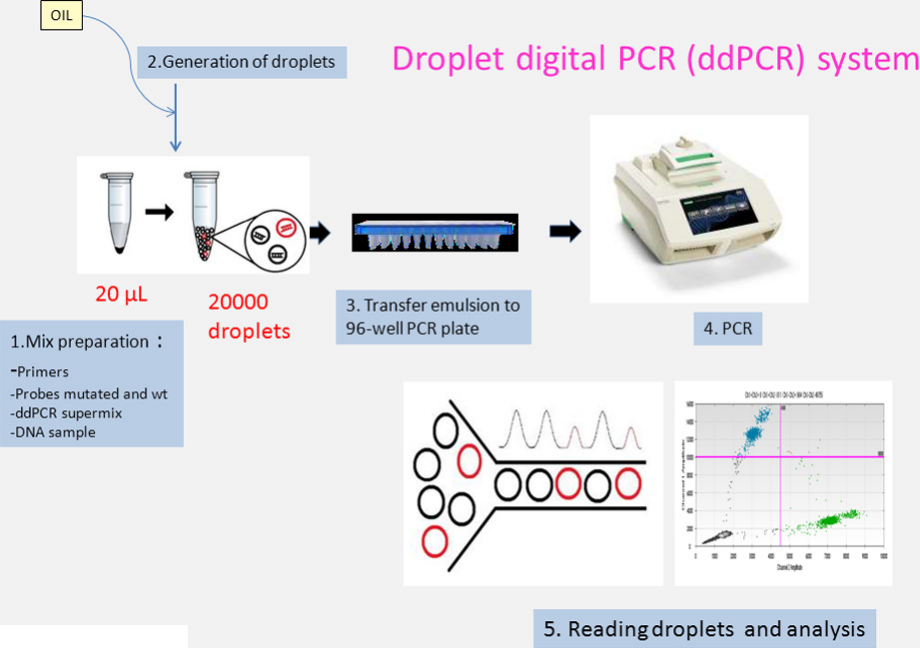
Illustration of different steps of droplet digital PCR (ddPCR) workflow.

The droplet digital PCR (ddPCR) workflow requires different steps involving droplet generation, the PCR procedure, and the droplet reading 13 (Fig.1). Five microliters of circulating DNA or 5 *μ*L of tumor DNA sample (10 ng/*μ*L) was added to 14 *μ*L of Droplet PCR Supermix (Bio-Rad Technologies, Marnes-la-Coquette, France) and 1 *μ*L of the primer/probe mixture. This 20 *μ*L sample was added to 70 *μ*L of Droplet Generation Oil (Bio-Rad Technologies) and used for droplet generation. Droplets were then thermal cycled as follows: 10 min at 95°C, 40 cycles of 94°C for 30 sec, 55°C (for *ALK* F1174L) or 62°C (for *ALK* R1275Q) for 1 min followed by 98°C for 10 min. Samples were then transferred to a QX100 digital droplet reader (Bio-Rad Technologies) for fluorescent measurement of FAM and VIC or HEX probes. Each sample was analyzed in duplicate. The primers and the probe targeting the hotspot F1174L (3520, T>C) were purchased from Biorad, whereas the primers and the probes specific for the hotspots F1174L (3522, C>A) and R1275Q (3824, G>A) were designed using Primer3Plus software and are described in Table1. They were used to a final concentration of 900 and 250 nmol/L, respectively. Each experiment contained positive and negative controls as well as no template controls. The cases showing a mutation were confirmed in a second experiment. Droplets were scored as positive or negative based upon their fluorescence intensity which was determined by gating a threshold using positive and negative controls as well as no template controls. Although in theory, one positive droplet should score the sample as positive, a minimal number of six positive droplets was defined to classify a sample as positive. A plasma sample was described as evaluable only if greater than 100 events (mutated+wild type [WT]) can be identified for each hotspot analyzed.

### Deep sequencing of *ALK* exon 23 and exon 25 regions with Illumina HiSeq2500 technology

Fifty nanograms of genomic DNA from each sample was amplified via a two-step PCR approach. In the first step, the *ALK* regions of interest containing F1174 and R1275 hotspots were amplified. The second PCR step consisted of addition of sample-specific barcodes for targeted resequencing in a single experiment. Amplicon sequencing (Illumina HiSeq2500, Eindhoven, the Netherlands) achieved an extremely high depth of coverage over the relevant hotspot (>80,000X). The background base variability (error rate) in 24 germline control samples was below 0.01% at the 3520, 3522, and 3824 position, respectively.

For each sample, the frequencies of the bases at the given position were then compared to those observed in the 24 controls. Statistical analyses were performed with the R statistical software (http://www.R-project.org). Fisher's exact two-sided tests were performed to compare percentages of bases between the data sets, that is, between a case and the controls.

### Statistical methods

The dichotomous results obtained by ddPCR from plasma and tumor samples were cross-tabulated. The sensitivity and the specificity were calculated according to the criteria published by Delacour et al. 22.

## Results

### *ALK* mutation detection in circulating DNA by ddPCR

**Figure 2:**
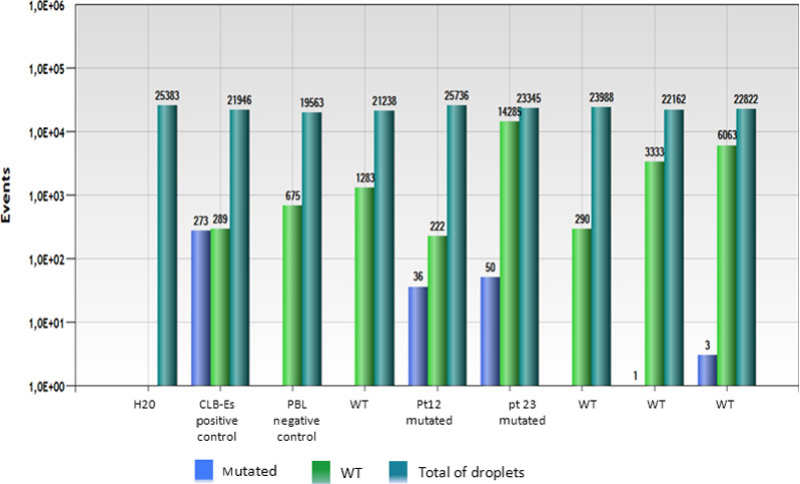
Representative bar graph of ddPCR analysis of plasma and control samples for identifying the R1275Q ALK mutation (the results of duplicates were merged). The number of droplets positive is indicated at the top of each bar (in blue = droplets containing mutated DNA, in green = droplets containing wild-type DNA). The bar in blue-green define the number of droplets analyzed (empty droplets + droplets containing wild or mutated DNA).

Among the 114 serum or plasma samples studied, 111 cases were described as evaluable. No mutation was detected in 87 samples, whereas 24 cases were found mutated (Table2) (Fig.2). Mutant to WT *ALK* ratio ranged from 0.15% to 43.7% and the prevalence of *ALK* mutations detected in circulating DNA samples by ddPCR was 21.62% (24/111). The mutation F1174L (exon 23 position 3520) and the mutation F1174L (exon 23 position 3522) were detected in 2 and 11 samples, respectively, whereas the mutation R1275Q (exon 25 position 3824) was identified in 15 samples. The mutations F1174L (exon 23 position 3522) and the mutation R1275Q were detected in the same sample in four cases.

**Table 2:** Results of F1174L and R1275Q *ALK* mutations detected by ddPCR in circulating DNA and tumor samples obtained from NB patients

		Plasma			Tumors						
			ddPCR				ddPCR			Hiseq sequencing	
	Stage	Event	Mutations (nucleotide substitution)	% alteration	Sample event	tumor cells (%)	Sanger sequencing Mutations (nucleotide substitution)	Mutations (nucleotide substitution)	% alteration	Mutations (nucleotide substitution)	% alteration
**PT1**^1^	4	D	F1174L (TTC>CTC)	0.15% (32/20,775)	BM D	80	WT	F1174L (TTC>CTC)	6.1% (595/9092)	F1174L (TTC>CTC)	7.20%
PT2^1^	4	D	F1174L (TTC>CTC)	21.6% (1361/4917)	—	—	NT	NT		NT	
**PT3**	4	D	F1174L (TTC>TTA)	1.54% (28/1796)	T D	80	WT	WT		WT	
**PT4**	3	D	F1174L (TTC>TTA)	5.3% (7/125)	T D	70	F1174L (TTC>TTA)	F1174L (TTC>TTA)	35.3% (3936/7222)	F1174L (TTC>TTA) **R1275Q (CGA>CAA)**	46.7% **0.2%**
**PT5**	4	D	F1174L (TTC>TTA)	1.5% (54/3553)	BM D	50	WT	WT		WT	
**PT6**	3	D	F1174L (TTC>TTA)	13.33% (22/143)	T D	80	F1174L (TTC>TTA)	F1174L (TTC>TTA) R1275Q (CGA>CAA)	16.7% (1667/8334) 1.2% (161/13440)	F1174L (TTC>TTA) R1275Q (CGA>CAA)	20.2% 2%
**PT7**	2B	D	F1174L (TTC>TTA)	5.26% (8/144)	T D	95	WT	WT		WT	
**PT8**	4	D	F1174L (TTC>TTA)	8.72% (15/157)	T D	80	F1174L (TTC>TTA)	F1174L (TTC>TTA)	34.6% (3750/7087)	F1174L (TTC>TTA)	38.50%
PT9	4	D	F1174L (TTC>TTA)	0.26% (34/12,960)	T D	90	F1174L (TTC>TTA)	NT		NT	
**PT10**	3	D	R1275Q (CGA>CAA)	9.4% (51/488)	T D	90	R1275Q (CGA>CAA)	R1275Q (CGA>CAA)	41% (2170/3110)	R1275Q (CGA>CAA)	46.70%
**PT11**	4	D	R1275Q (CGA>CAA)	7.3% (51/643)	T D	90	WT	F1174L (TTC>TTA) R1275Q (CGA>CAA)	1.7% (244/13813) 6.5% (48/680)	F1174L (TTC>TTA) R1275Q (CGA>CAA)	2.3% 8.9%
PT12	4	D	R1275Q (CGA>CAA)	14% (36/222)	T D	50	NT	R1275Q (CGA>CAA)	27.4% (1305/3450)	NT	
PT13	4	D	R1275Q (CGA>CAA)	4% (551/13245)	—	—	NT	NT		NT	
**PT14**	4	D	R1275Q (CGA>CAA)	0.5% (39/7771)	T PC	70	R1275Q (CGA>CAA)	R1275Q (CGA>CAA)	20.5% (1381/5343)	**F1174L (TTC>TTA)** R1275Q (CGA>CAA)	**0.1%** 24.6%
**PT15**^1^	4	D	R1275Q (CGA>CAA)	26% (71/202)	T PC	80	R1275Q (CGA>CAA)	R1275Q (CGA>CAA)	19.9% (1836/7003)	R1275Q CGA>CAA)	26.80%
**PT16**	4	D	R1275Q (CGA>CAA)	10.9% (54/440)	T D	90	R1275Q (CGA>CAA)	R1275Q (CGA>CAA)	38.3% (5717/9213)	R1275Q (CGA>CAA)	47.30%
**PT17**^1^	**4**	D	R1275Q (CGA>CAA)	34.3% (474/909)	T PC	85	R1275Q (CGA>CAA)	R1275Q (CGA>CAA)	18.3% (2815/12526)	R1275Q (CGA>CAA)	23.50%
**PT18**	4	D	R1275Q (CGA>CAA)	1% (13/1225)	T D	95	R1275Q (CGA>CAA)	R1275Q (CGA>CAA)	27.6% (3250/8517)	R1275Q (CGA>CAA)	34.30%
**PT19**^1^	4	D	R1275Q (CGA>CAA)	43.7% (518/617)	T D	90	R1275Q (CGA>CAA)	R1275Q (CGA>CAA)	40.8% (6460/9359)	R1275Q (CGA>CAA)	49.70%
PT20	4	D	R1275Q (CGA>CAA)	0.15% (16/10358)	—	—	NT	NT		NT	
**PT21**	**4**	D	F1174L (TTC>TTA) R1275Q (CGA>CAA)	1.21% (18/1470) 1.7% (34/1962)	T D	80	WT	F1174L (TTC>TTA) R1275Q (CGA>CAA)	2.3% (437/18426) 2.6% (26/989)	F1174L (TTC>TTA) R1275Q (CGA>CAA)	9.1% 2.6%
					LN D	70	R1275Q (CGA>CAA)	NT for F1174L R1275Q (CGA>CAA)	20.2% (136/537)	F1174L (TTC>TTA) R1275Q (CGA>CAA)	0.37% 23.9%
**PT22**	3	D	F1174L (TTC>TTA) R1275Q (CGA>CAA)	3.13% (6/186) 3.6% (7/186)	T D	80	R1275Q (CGA>CAA)	F1174L (TTC>TTA) R1275Q (CGA>CAA)	0.07% (8/10022) 40.1% (4310/6222)	F1174L (TTC>TTA) R1275Q (CGA>CAA)	0.05% 46.7%
PT23	4	D	F1174L (TTC>TTA) R1275Q (CGA>CAA)	33.64% (5179/10,216) 0.35% (50/14,285)	T D	80	F1174L (TTC>TTA)	F1174L (TTC>TTA)	32.7% (3667/7538)	F1174L (TTC>TTA)	37.30%
					T PC	80	F1174L (TTC>TTA)	NT for F1174L WT for R1275Q		F1174L (TTC>TTA)	24.60%
PT24	4	D	F1174L (TTC>TTA) R1275Q (CGA>CAA)	3.2% (79/2412) 0.7% (9/1265)	T D	ND	WT	R1275Q (CGA>CAA)	1% (7/707)	R1275Q (CGA>CAA)	0.16%

Comparison with Sanger sequencing and deep sequencing. D, diagnosis, PC, post chemotherapy, T, tumor, BM, bone marrow, LN, lymph node, WT, wild type, M, mutated, NT, not tested.

1 Patients for whom a cell line was established 3. The discordances between ddPCR and sequencing procedures on tumor samples are indicated in bold. The numbers in brackets in columns “alteration” = number of droplets containing mutated DNA/number of droplets containing wild-type DNA.

### *ALK* mutation detection in tumor samples by Sanger sequencing and ddPCR and Illumina Deep sequencing

Among the 111 cases previously evaluable by ddPCR on circulating DNA, the Sanger sequencing was performed on 101 DNA tumor samples containing more than 50% of malignant cells. The 10 other tumor samples were not available or containing a low proportion of tumor cells. Among the 101 tumors studied, the mutation F1174L (exon 23) was identified in five cases and the mutation R1275Q (exon 25) was detected in nine cases. All *ALK* mutations detected by Sanger sequencing were also identified in circulating DNA. All *ALK* mutations were somatic since no mutation was detected in corresponding normal DNA samples (data not shown). The proportion of mutated cases after analysis of DNA tumors samples by Sanger sequencing was lower than the ratio of mutated cases detected by ddPCR in plasma samples. Furthermore, some mutations present in the plasma of patients were also previously identified on cell lines established from the same patient (patients’ nos. 1, 2, 15, 17, and 19) 3. For these different reasons, we decided to study DNA obtained from tumor samples using ddPCR system since this technology was described to be highly sensitive. Among the 101 tumors studied by Sanger sequencing, 60 samples (40 cases defined as WT for the two hotspots and 20 samples defined mutated by ddPCR analysis on circulating DNA samples) were selected and analyzed. ddPCR confirmed the mutations identified by Sanger sequencing but also identified eight additional mutations, four were the mutation F1174L on exon 23 (one was specific of position 3520 and three were specific of position 3522) and four involved the mutation R1275Q on exon 25. Overall, a total of 17 patients showed one or two *ALK* mutations after analysis of DNA tumor samples by ddPCR (Table2). In 40 tumor samples defined as WT after analysis of circulating DNA, no mutation was found in the hotspots of the exons 23 or 25. Mutant to WT *ALK* ratio was ranged from 16.7% to 41% for the cases identified by the ddPCR system and Sanger sequencing, whereas it decreased to 0.07% for the samples detected only by ddPCR.

To validate the data obtained by ddPCR, the tumors of patients showing *ALK* mutations detected in plasma or tumor samples were analyzed by deep sequencing using Illumina HiSeq2500 technology in a blinded study. A good concordance was observed between both techniques although for two cases, mutations were only detected by deep sequencing (PT4 (R1275Q, 3824,G>A) and PT14 (F1174L(3522,C>A) (Tables2 and 3).

**Table 3:** Base frequencies at the coordinates of interest in NB samples analyzed by deep sequencing (Illumina HiSeq 2500)

Chr position/patient ID	Reads	T		G		C		A	
		%	*P*-value	%	*P*-value	%	*P* value	%	*P* value
F1174L 3520 T>C, chr2:29220831									
Controls	79,7051	99.987	NA	0	NA	0.01	NA	0.003	NA
PT1	32,757	92.805	0.00E+00	0	NA	**7.192**	0.00E+00	0.003	NA
PT3	30,624	9999	NA	0	NA	0.01	NA	0	NA
PT4	32,998	99.979	NA	0.003	NA	0.009	NA	0.009	NA
PT5	35,142	99.98	NA	0	NA	0.017	NA	0.003	NA
PT6	34,977	99.977	NA	0.003	NA	0.011	NA	0.009	NA
PT7	37,846	99.976	NA	0	NA	0.021	NA	0.003	NA
PT8	37,000	99.986	NA	0	NA	0.011	NA	0.003	NA
PT10	31,101	99.987	NA	0.003	NA	0.006	NA	0.003	NA
PT11	18,320	99.989	NA	0,005	NA	0.005	NA	0	NA
PT14	31,871	99.991	NA	0.003	NA	0.006	NA	0	NA
PT15	44,349	99.982	NA	0.002	NA	0.014	NA	0.002	NA
PT16	35,523	99.989	NA	0.003	NA	0.006	NA	0.003	NA
PT17	31,232	99.99	NA	0	NA	0.01	NA	0	NA
PT18	34,764	99.974	NA	0	NA	0.023	NA	0.003	NA
PT19	36,172	99.981	NA	0	NA	0.014	NA	0.006	NA
PT21	35,015	99.991	NA	0	NA	0.009	NA	0	NA
PT21	22,403	99.987	NA	0	NA	0.004	NA	0.009	NA
PT22	42,150	99.988	NA	0	NA	0.009	NA	0.002	NA
PT23	36,548	99.984	NA	0	NA	0.014	NA	0.003	NA
PT23	37,033	99.989	NA	0	NA	0.005	NA	0.005	NA
PT24	31,343	99.987	NA	0	NA	0.01	NA	0.003	NA
F1174L 3522, C>A, chr2:29220829									
Controls	796,257	0.009	NA	0.003	NA	99.976	NA	0.011	NA
PT1	32,732	0.009	NA	0.003	NA	99.985	NA	0.003	NA
PT3	30,566	0.003	NA	0	NA	99.977	NA	0.02	NA
PT4	32,908	0.015	NA	0.006	NA	53.27	0.00E+00	**46.709**	0.00E+00
PT5	35,113	0.014	NA	0	NA	99.974	NA	0.011	NA
PT6	34,885	0.011	NA	15.055	0.00E+00	64.718	0.00E+00	**20.212**	0.00E+00
PT7	37,824	0.003	NA	0	NA	99.989	NA	0.008	NA
PT8	36,884	0.005	NA	0.005	NA	61.463	0.00E+00	**38.521**	0.00E+00
PT10	31,071	0.003	NA	0	NA	99.99	NA	0.006	NA
PT11	18,266	0.011	NA	0	NA	97.64	0.00E+00	**2.349**	0.00E+00
PT14	31,840	0.009	NA	0	NA	99.846	3.37E-16	**0.144**	1.66E-25
PT15	44,301	0.007	NA	0	NA	99.989	NA	0.002	NA
PT16	35,485	0.014	NA	0.003	NA	99.969	NA	0.011	NA
PT17	31,207	0	NA	0.003	NA	99.99	NA	0.006	NA
PT18	34,734	0.014	NA	0.003	NA	99.977	NA	0.006	NA
PT19	36,146	0.019	NA	0.011	NA	99.967	NA	0.003	NA
PT21	34,986	0.009	NA	0.003	NA	90.896	0.00E+00	**9.089**	0.00E+00
PT21	22,338	0.004	NA	0	NA	99.624	1.04E-56	**0.367**	1.16E-74
PT22	42,101	0.012	NA	0.002	NA	99.929	2.70E-01	**0.05**	3.41E-02
PT23	36,413	0.014	NA	0.003	NA	62.722	0.00E+00	**37.259**	0.00E+00
PT23	36,925	0.008	NA	0	NA	75.355	0.00E+00	**24.636**	0.00E+00
PT24	31319	0.003	NA	0	NA	99.987	NA	0.006	NA
R1275Q 3824 G>A, chr2:29209798									
Controls	1,005,867	0.016	NA	99.956	NA	0.001	NA	0.027	NA
PT1	49,218	0.008	NA	99.974	NA	0	NA	0.018	NA
PT3	53,946	0.011	NA	99.972	NA	0.002	NA	0.015	NA
PT4	51,659	0.006	NA	99.783	1.41E-31	0	NA	**0.211**	3.33E-46
PT5	73,384	0.022	NA	99.95	NA	0.001	NA	0.027	NA
PT6	68,439	0.013	NA	98.004	0.00E+00	0.001	NA	**1.981**	0.00E+00
PT7	49,685	0.002	NA	99.964	NA	0.002	NA	0.032	NA
PT8	52,130	0.006	NA	99.969	NA	0	NA	0.025	NA
PT10	50,187	0.01	NA	53.265	0.00E+00	0.004	NA	**46.715**	0.00E+00
PT11	46,445	2.119	0.00E+00	88.937	0.00E+00	0	NA	**8.942**	0.00E+00
PT14	27,110	0	NA	75.393	0.00E+00	0.007	NA	**24.592**	0.00E+00
PT15	34,878	0.011	NA	73.195	0.00E+00	0.003	NA	**26.788**	0.00E+00
PT16	47,751	0.013	NA	52.667	0.00E+00	0.004	NA	**47.31**	0.00E+00
PT17	56,685	0.023	NA	76.465	0.00E+00	0	NA	**23.512**	0.00E+00
PT18	52,198	0	NA	65.732	0.00E+00	0.004	NA	**34.262**	0.00E+00
PT19	55,451	0.005	NA	50.333	0.00E+00	0.002	NA	**49.651**	0.00E+00
PT21	51,696	0.006	NA	97.373	0.00E+00	0	NA	**2.621**	0.00E+00
PT21	44,126	0.005	NA	76.069	0.00E+00	0	NA	**23.925**	0.00E+00
PT22	48,821	0.006	NA	53.327	0.00E+00	0	NA	**46.662**	0.00E+00
PT23	52,852	0.011	NA	99.97	NA	0	NA	0.019	NA
PT23	59,037	0.007	NA	99.978	NA	0	NA	0.015	NA
PT24	51,971	0.01	NA	99.835	1.59E-16	0	NA	**0.156**	9.57E-26

The mutations detected by deep sequencing and ddPCR are indicated in bold.

### Comparison between the data obtained on circulating DNA and DNA tumor samples after ddPCR analysis

We found a perfect concordance for the analysis of F1174L mutation (3520, T>C) between circulating DNA and DNA tumor samples. Indeed, one case was positive in blood and the tumor (PT1) and the other cases were negative in DNA tumor samples and circulating DNA samples. For the F1174L mutation (3522, C>A), in four cases (patients’ nos. 3, 5, 7, and 24) this mutation was detected in blood but was not found in the tumor, whereas one patient (no. 11) showed this mutation only in tumor sample. For the 55 other cases, this hotspot was defined as WT for 49 patients and mutated for six patients in both circulating DNA and DNA tumors. Finally, when we analyzed the hotspot R1275Q, 46 and 12 cases were found WT and mutated, respectively, in both circulating DNA and DNA tumor samples, one case showed this mutation only in the tumor (no. 6), whereas for one other case the mutation was detected only in the blood (no. 23) Overall, the sensitivity of our test for the detection of F1174L mutations to position (3520, (T>C) and 3522 (C>A)) and R1275Q mutation was 100%, 85.7%, and 92.3%, respectively, and the specificity was 100%, 92.4%, and 97.9%, respectively. The specificity and the sensitivity of our assay may have been underestimated since (1) the tumors analyzed in this study were microbiopsies obtained from primary tumor or metastatic site, and (2) the tumors may be heterogeneous as shown recently 23. This last point was confirmed by the analysis of the primary tumor (80% of malignant cells) and a lymph node containing 70% of tumor cells for the patient no. 21. The ratio of R1275Q mutation was 2.6% and 20.2% in the primary tumor and the lymph node, respectively.

## Discussion

In this report, we present the feasibility of screening for the presence of two hotspot *ALK* mutations in NB patients by analysis of circulating DNA obtained from 200 *μ*L of serum or plasma samples collected at diagnosis using ddPCR system. The basis for successful circulating DNA detection is the selection of an isolation method that ensures extraction of a sufficient amount of quality DNA. As shown by different teams 24,25, the choice of commercial kits is important and QIAampDNA Micro Kit and QIAamp MinElute Virus Vacuum Kit (Qiagen) were described as more suitable. We have confirmed these results (data not shown). It was crucial for our study to use a minimal quantity of blood, as it is difficult to collect a large volume of blood since the patients with NB tumors are young (age often below 7 years with a majority being between 2 and 5 years). To our knowledge, this is the first study showing the detection of rare mutant alleles from only few milliliters (200 *μ*L) of serum or plasma. Overall, circulating DNA was isolated from 114 patient blood samples and interpretable in 111 cases. The *ALK* mutation was identified in 21.62% of patients. This high frequency of *ALK* mutations is consistent with the previous study published by Sausen et al. in which 10 cases showed *ALK* mutation among the 41 NB samples analyzed by next-generation sequencing analysis 26.

Our aim was to compare the data obtained from circulating DNA to the data obtained by Sanger sequencing (exon 23 and exon 25) of tumor DNAs. Among the 111 cases, the Sanger sequencing was possible in 101 tumor DNA samples containing >50% tumor cells. *ALK* mutation was identified in 14 cases (13.8%). All *ALK* mutations detected by Sanger sequencing were also identified in circulating DNA. However, a large number of mutations found in plasma were not identified by Sanger sequencing. This technique is described to be less sensitive (20%) 27 than the ddPCR system 13. Therefore, the ddPCR was used to analyze DNA tumors obtained from 60 specimens. Additional mutations were found with this procedure and the results obtained by ddPCR were validated using a deep sequencing method. The codetection of two mutations was only detectable with highly sensitive methods. The presence of two different mutations in the same patient has been previously reported 4,5. The heterogeneity of tumors can explain the detection of these two mutations found in different subclones present in the same NB sample or in different tumor specimens (primary tumor or distant metastases) found in the same patient. Among the 24 patients shown in Table2, different cases illustrate well these suggestions. For the patient no. 21, the primary tumor containing 80% of tumor cells showed 2.6% of R1275Q mutation, whereas this mutation was found at a ratio of 20.2% in lymph node showing 70% of tumor cells. This case shows that the distribution of the mutated population varies among different tumor sites. Chan et al. have shown that cell-free DNA fragments from multiple lesions in the same individual all mix together in the peripheral blood 28. The codetection of two mutations was also observed in circulating DNA samples therefore ddPCR mutation detection in free circulating DNA may overcome issues of tumor heterogeneity and detect rare mutations which might not be identified by studying a single site of disease. For example, in the patient no. 23, both F1174L (exon 23 position 3522) and R1275Q mutations are detected in plasma sample but only the F1174L mutation was found in primary tumor. For this case, the R1275Q was not detected on primary tumor at diagnosis or after chemotherapy but perhaps this mutation was present on metastatic sites. Unfortunately, no biopsy or puncture obtained from metastatic sites was available.

To our knowledge, this retrospective study is the first study evaluating the feasibility of ddPCR to identify the presence of hotspot *ALK* mutations in NB patients by analysis of free circulating DNA. To identify the other *ALK* mutations specific of NB tumors as detected for patients PT6 (3522, C>G) and PT11 (3824, G>T) by deep sequencing (Table3), other ddPCR assays are currently in development. New protocols based on ALK-targeted therapy by crizotinib or other ALK targeting molecules have opened for the treatment of NB patients in relapse if their tumors showed mutation and/or amplification of the *ALK* gene and new tumor sampling is strongly recommended at the time of relapse 23 for evaluating *ALK* mutational status given the observation that an emergence of *ALK* mutation might be seen at relapse. As biopsy or puncture of the tumor at this time of the disease is not always possible or does not permit to collect enough material for deep sequencing analysis, the study of free circulating DNA by ddPCR appears as a reliable, noninvasive blood test to assess *ALK* mutational status at F1174 and R1275 hotspots. Currently, it is too early to know whether crizotinib will be effective in children who harbor somatic *ALK* mutations 12. The assays performed in vitro and in xenograft models have shown that the two mutations, R1275Q and F1174L found in NB tumors, display a different sensitivity to crizotinib, the first being responder to crizotinib and the latter being resistant 10. The reduced susceptibility of F1174L mutated *ALK* to crizotinib inhibition results from an increase in ATP binding affinity, suggesting that higher doses of crizotinib may mitigate the resistance. Another promising strategy using PI3K/AKT/mTOR pathway inhibitors in combination with crizotinib has been suggested recently for the treatment of NB patients showing *ALK*^F1174L^ mutation 29. In consequence, the treatment should be conducted according to *ALK* mutational status. The analysis of circulating DNA by ddPCR should help clinicians to identifying the patients who might benefit from ALK-targeted therapy and to defining which treatment should be performed.
