# Erratum

**DOI:** 10.1002/cam4.637

**Published:** 2016-02-02

Combaret, V., Iacono, I., Bellini, A., Bréjon, S., Bernard, V., Marabelle, A., Coze, C., Pierron, G., Lapouble, E., Schleiermacher, G., and Blay, J. Y., (2015). Detection of tumor *ALK* status in neuroblastoma patients using peripheral blood *Cancer Medicine,* 4 (4), 540–550. doi: 10.1002/cam4.414

In the article which appeared in Cancer Medicine entitled ‘Detection of tumor *ALK* status in neuroblastoma patients using peripheral blood’, the author would like to correct caption of figure 1.

The current caption is:

Illustration of different steps of droplet digital PCR (ddPCR) workflow.

It should read as:

Illustration of different steps of droplet digital PCR (ddPCR) workflow. This figure was adapted by permission from the American Association for Cancer Research: Oxnard GR, Paweletz CP, Kuang Y, Mach SL, O'Connell A, Messineo MM, Luke JJ, Butaney M, Kirschmeier P, Jackman DM, Jänne PA. Noninvasive detection of response and resistance in EGFR‐mutant lung cancer using quantitative next‐generation genotyping of cell‐free plasma DNA. Clin Cancer Res. 2014 Mar 15; 20(6):1698‐705. doi: 10.1158/1078‐0432.CCR‐13‐2482.

We apologize for the error.

